# Nasal delivery of killed *Bacillus subtilis* spores protects against influenza, RSV and SARS-CoV-2

**DOI:** 10.3389/fimmu.2025.1501907

**Published:** 2025-04-02

**Authors:** Rong Xu, Huynh A. Hong, Shadia Khandaker, Murielle Baltazar, Noor Allehyani, Daan Beentjes, Tessa Prince, Yen-Linh Ho, Linh Hanh Nguyen, Daniel Hynes, William Love, Simon M. Cutting, Aras Kadioglu

**Affiliations:** ^1^ Department of Clinical Infection, Microbiology and Immunology, University of Liverpool, Liverpool, United Kingdom; ^2^ SporeGen Ltd., London Bioscience Innovation Centre, London, United Kingdom; ^3^ Department of Clinical Laboratory Sciences, College of Applied Medical Sciences, Shaqra University, Riyadh, Saudi Arabia; ^4^ Department of Infection Biology and Microbiomes, University of Liverpool, Liverpool, United Kingdom; ^5^ Huro Biotech Joint Stock Company, Ho Chi Minh, Vietnam; ^6^ Destiny Pharma Plc., Sussex Innovation Centre, Brighton, United Kingdom

**Keywords:** *Bacillus* spores, SARS-CoV-2, influenza, respiratory syncytial virus (RSV), mucosal immunity

## Abstract

**Introduction:**

Spores of the bacterium *Bacillus subtilis* (*B. subtilis*) have been shown to carry a number of properties potentially beneficial for vaccination. Firstly, as vehicles enabling mucosal delivery of heterologous antigens and secondly, as stimulators of innate immunity. Here, we have examined the specificity of protection conferred by the spore-induced innate response, focusing on influenza H1N1, respiratory syncytial virus (RSV), and coronavirus-2 (SARS-CoV-2) infections.

**Methods:**

*In vivo* viral challenge murine models were used to assess the prophylactic anti-viral effects of *B. subtilis* spores delivered by intranasal instilling, using an optimised three-dose regimen. Multiple nasal boosting doses following intramuscular priming with SARS-CoV-2 spike protein was also tested for the capability of spores on enhancing the efficacy of parenteral vaccination. To determine the impact of spores on immune cell trafficking to lungs, we used intravascular staining to characterise cellular participants in spore-dosed pulmonary compartments (airway and lung parenchyma) before and after viral challenge.

**Results:**

We found that mice pre-treated with spores developed resistance to all three pathogens and, in each case, exhibited a significant improvement in both survival rate and disease severity. Intranasal spore dosing expanded alveolar macrophages and induced recruitment of leukocyte populations, providing a cellular mechanism for the protection. Most importantly, virus-induced inflammatory leukocyte infiltration was attenuated in spore-treated lungs, which may alleviate the associated collateral tissue damage that leads to the development of severe conditions. Remarkably, spores were able to promote the induction of tissue-resident memory T cells, and, when administered following an intramuscular prime with SARS-CoV-2 spike protein, increased the levels of anti-spike IgA and IgG in the lung and serum.

**Conclusions:**

Taken together, our results show that *Bacillus* spores are able to regulate both innate and adaptive immunity, providing heterologous protection against a variety of important respiratory viruses of high global disease burden.

## 1 Introduction

Lower respiratory tract infections (LRTIs), including seasonal flu caused by influenza viruses and most recently, coronavirus disease (COVID-19), represent a significant global health burden. Prior to the emergence of the COVID-19 pandemic, LRTIs were responsible for over two million deaths annually and were the leading cause of morbidity and mortality worldwide (1). Since the onset of the COVID-19 outbreaks in January 2020 however, the impact of LRTIs on global health has escalated significantly. As of December 2023, COVID-19 alone has resulted in more than seven million deaths worldwide. Despite the focus on COVID-19, seasonal flu continues to pose a substantial threat to public health. For example, since May 2022 Australia has seen a rapid rise in influenza A viral infections indicating a potential resurgence of seasonal flu epidemics (2). Furthermore, co-circulation of influenza and COVID-19 may exacerbate illness severity, further straining public healthcare systems. Vaccination remains one of the most successful public health interventions for preventing infectious diseases and reducing global disease burden. However, RNA viruses such as influenza and SARS-CoV-2 mutate rapidly and continuously acquire antigenically relevant substitution in key viral proteins. This has resulted in the continued emergence of new variants of concern (VoCs) that can infect vaccinated individuals. Novel prophylactic strategies are therefore required for a more effective control of the recurrent waves of COVID-19 and influenza infection and to pre-empt the next pandemic.

*Bacillus* spores are dormant bacterial life forms typically found in the environment (e.g., soil and dust) (3). As particularly robust entities they can withstand extremes of temperature, desiccation and exposure to noxious chemicals, yet under favourable conditions can germinate and resume growth. *Bacillus* spores have been used primarily for mucosal vaccination utilising the proteinaceous layer of the spore coat for delivery of heterologous antigens (4, 5). A further aspect of the spores is their ability to activate innate immune responses, e.g., promoting dendritic cell maturation and natural killer (NK) cell recruitment (6), which may assist host defence against microbial infections through heterologous immunity.

Mechanistically, heterologous immunity is driven by cross-reactive T cell responses and innate immune imprints. Cross-reactive T cells can recognise pathogens that share antigenic determinants with previously encountered pathogens. For example, CD8^+^ T cells from lymphocytic choriomeningitis virus (LCMV)-immunised mice can also respond to pichinde virus (PV) and vaccinia virus (VV) infections (7). Between different classes of pathogens, however, heterologous immunity is more commonly elicited through bystander T cell activation and innate immune cells. This can be illustrated with the current BCG (*Bacillus* Calmette-Guerin) TB vaccine (this being live attenuated *Mycobacterium bovis*), which has been shown to enhance the activation of monocytes and NK cells against subsequent infections by malaria, *Candida albicans* and *Staphylococcus aureus* (8, 9).

*Bacillus* spores have been investigated as vaccine vectors or adjuvants in previous studies. We first found that spores may by themselves possess anti-viral properties when testing killed spores as a mucosal adjuvant against influenza H5N1 viral infection in BALB/c mice (6). A follow up mouse study showed that live spores were able to activate alveolar macrophages which enhanced their anti-viral activity against RSV (10). *Bacillus* species have been used as human probiotics to improve gut health. More recently, the safety and potential health benefits of probiotic live *Bacillus* spores by nasal spraying were tested, as an addition to the standard care, in paediatric patients with symptomatic influenza infection (11). However, no study so far has analysed in detail how intranasal dosing of spores affects immune cell trafficking in lungs before and after viral challenge or whether it impacts the development of adaptive immunity. Moreover, despite a couple of studies exploring the potential of spores as a carrier for COVID-19 vaccine, it remains unknown whether the heterologous immunity elicited by spores at the mucosal site could protect against SARS-CoV-2 infection either by themselves alone or following parental vaccination.

In this study, we demonstrate that intranasal administration of heat-killed *B. subtilis* spores using a universal dosing regimen provides protection against three major respiratory viruses, influenza H1N1, SARS-CoV-2 (Beta and Omicron variants) and RSV, strongly suggesting a universal anti-viral effect of spores. Spore dosing not only recruited various immune cells, including neutrophils, NK cells and T cells, but also promoted the expansion of lung-resident alveolar macrophages. Interestingly, spore-treated lungs were less responsive to virus-induced immune cell infiltration, suggesting that spores may confer protection by inducing tolerance to the detrimental inflammatory responses activated by the viral infections. Importantly, increased levels of tissue-resident memory T cells (TRMs) were detected in the airway of spore-dosed lungs following viral infection and intranasal spore dosing following intramuscular immunisation with SARS-CoV-2 Spike protein was able to boost the production of both anti-spike IgG and IgA antibodies. This indicates that spores have the potential to ‘pull’ pre-existing immune memory to mucosal sites if used in conjunction with current COVID-19 and/or flu vaccines for example. Our results suggest that heat-killed *B. subtilis* spores have the potential to be developed as a nasal Pan-Pneumovirus vaccine.

## 2 Materials and methods

### 2.1 Production of *Bacillus* spores

*B. subtilis* strain DSM 32444 was used for this study. This strain is recommended as safe for human consumption (USA FDA GRAS-notification GRN 000905). Spores were prepared in batch culture (200 mL) and suspensions in dH_2_O inactivated by autoclaving (121°C, 20 min., 15 psi) and are referred to henceforth as DSM 32444^K^. Validation of spore inactivation was made by serial dilution of heat-treated spore suspensions and plating for viability on agar growth medium and with no resulting bacterial growth meeting the required standard. Aliquots (1 mL) were used for animal studies with each lot containing 5 x 10^10^ inactivated spore particles (determined by microscopic counting using a Neubauer counting chamber).

### 2.2 Viruses

H1N1 (A/PR/8 strain) viral stocks were kindly provided by Dr John W McCauley (and Dr Michael Bennett) from the Francis Crick Institute. The SARS-CoV-2 strains used in this study, Beta variant (Bristol/Liverpool isolate) and Omicron variant BA.1, were grown from the stocks of Prof. Julian Hiscox (University of Liverpool). Virus stocks were propagated using Vero E6 cells and viral titres determined by plaque assays. Work with SARS-CoV-2 was performed at containment level 3, following risk assessments and standard operating procedures approved by the University of Liverpool Biohazards Sub-Committee and by the UK Health and Safety Executive. The respiratory syncytial virus was a kind gift from Prof. Jürgen Schwarze (University of Edinburgh), and the viral stocks were produced and maintained in our lab.

### 2.3 Mice

Wildtype C57BL/6J, BALB/c, and K18-hACE2 transgenic mice were purchased from Charles River. All mice used in this study were 7-10 weeks and age-matched animals were randomly assigned to experimental groups. All mice were bred and maintained in individually ventilated cages at 22 ± 1°C and 65% humidity with a 12hr light-dark cycle. Prior to use, mice were acclimatised for one week with free access to food and water. All experimental protocols were approved and performed in accordance with the regulations of the Home Office Scientific Procedures Act (1986), project licence P86DE83DA and the University of Liverpool Ethical and Animal Welfare Committee.

### 2.4 Spore dosing and viral infection

Mice were lightly anaesthetised with O_2_/isoflurane and dosed intranasally (i.n.) with 2 or 3 doses of spores (1.5 × 10^9^/30μL per dose, once per week). 7 days (or 27 days for Omicron infection) post-last dose of spores, mice were i.n. challenged with 50μL of virus inoculum. For survival experiment, infected mice were monitored and scored regularly for signs of disease (12). Mice that became lethargic were humanely culled with a rising concentration of CO_2_ and counted as death events. Bronchoalveolar lavage (BAL) and lung tissues were harvested at indicated timepoints for the analysis of viral loads and immune responses. BAL fluid was collected as previously described (13), by inserting a catheter into the trachea and instilling 2 × 1mL of cold Hank’s balanced salt solution (HBSS, Sigma Aldrich) with 100μM of EDTA (Fisher Scientific). Cytokine levels in the BAL were measured using LEGENDplex Mouse Anti-Virus Response Panel (13-plex) (BioLegend), following the manufacturer’s instructions.

### 2.5 Lung tissue processing

Lung immune cells were isolated as previously described (14). Briefly, lung tissues were cut into pieces and digested with 300U/ml of type 1 collagenase (Gibco) and 75μg/mL of DNase 1 (Sigma Aldrich). Digested lung pieces were then mechanically dissociated by pushing through a 70μM cell strainer and then subjected to red blood cell lysis to generate single cell suspension for restimulation and flow cytometry staining.

Isolated lung cells from each animal were counted for total live cells based on AO/PI staining. FACS staining was carried out to determine the proportions of immune cell subsets in the whole cell population. Lung cell numbers were calculated based on the total cell count and the proportion of each immune cell subset. BAL cell numbers were determined by acquiring all cell events on the flow cytometry after staining.

For viral quantification, the lung lobes were dissociated by an electronic tissue homogeniser (Ultra Turrax, IKA) for H1N1 and RSV infected lungs. For SARS-CoV-2 infected animals, the left lung lobe was collected in Precellys beads tubes (Bertin Technologies) filled with 1mL of TRIzol reagent and homogenised using a Bead Ruptor 24 (Omni International).

### 2.6 Quantification of virus

The viral loads in the lung of H1N1 infected animals were determined by 50% tissue culture infectious dose (TCID_50_) in Madin-Darby canine kidney (MDCK) cells (ATCC, CCL-34) (15).

Immunoplaque assay was used to quantify RSV infected lungs (16). Briefly, serial diluted lung tissue homogenates were inoculated onto HEp-2 cells (ATCC, CCL-23). Viral plaques were stained by monoclonal goat anti-RSV antibody (ABD serotec, UK), followed by a secondary extravidin peroxidase conjugate and an Amino-ethylcarbazole substrate (both obtained from Sigma-Aldrich, UK). The number of viruses were expressed as plaque forming units (PFU) per 1 mL of HEp-2 cell supernatant.

For SARS-CoV-2 infected mice, RNA was extracted from the TRIzol lysed lungs, and viral RNA levels were measured using the Luna® Universal Probe One-Step RT-qPCR Kit (New England Biolabs) following the manufacturer’s instructions. WHO International Standard for SARS-CoV-2 RNA (inactivated England/02/2020 isolate, NIBSC 20/146) were used to produce a standard curve for the quantification of absolute viral copies. qPCR was performed on Bio-Rad CFX96™ using 2019-nCoV N1 primer and probe published by the United States Centres for Disease Control and Prevention (U.S. CDC), the sequences (5’-3’) are as below.

2019-nCoV N1-Forward: GACCCCAAAATCAGCGAAAT
2019-nCoV N1-Reverse: TCTGGTTACTGCCAGTTGAATCTG
2019-nCoV N1-Probe: [6FAM]ACCCCGCATTACGTTTGGTGGACC[BHQ1]

### 2.7 Flow cytometry

Single cells were incubated with Live/Dead Fixable Aqua Stain (Fisher Scientific) at 1:1000 to determine viable cells. Cell surface staining was performed with fluorochrome-conjugated anti-mouse monoclonal antibodies (mAbs) in the presence of True-Stain Monocyte Blocker (1:20) (BioLegend). mAbs used in this study including CD45-PE-Cy7 (30-F11), CD3-FITC (17A2), CD11b-PE (M1/70), Ly6G-APC-Cy7 (1A8), NKp46-APC (29A1.4), CD8-PE-Cy7 (53-6.7), CD103-PE (QA17A24), CD69-APC (H1.2F3) from BioLegend; SiglecF-BV421 (E50-2440) from BD Biosciences; and CD4-VioBlue (REA604) from Miltenyi Biotec. Stained samples were acquired on BD FACSCanto II and analysed using Flowjo 10.4.

### 2.8 Histology

Lungs were dissected and fixed with 10% (v/v) neutral buffered formalin (Sigma Aldrich) and sent to LBIH Biobank, Liverpool for embedding, sectioning and haematoxylin and eosin (H&E) staining. Stained sample slides were scanned and examined using QuPath-0.2.3.

### 2.9 Determination of antibody responses by indirect ELISA

Serum and lung samples taken at day 49 (serum) and 50 (lungs) were taken from animals that had been primed (i.m.) with recombinant SARS-CoV-2 Spike protein (50 μL, 5 μg) of formulated protein (Wuhan variant; SinoBiological (Cat Nos. 40589-V08B1) in each hind quadricep muscle) and then boosted (i.n.) with DSM 32444^K^ spores (1 X 10^9^ CFU; 10 μL, 5 μL/nare). Antibody (anti-Spike) levels in serum (IgG) and lungs (IgA) were determined by indirect ELISA as described elsewhere (17).

### 2.10 Statistical analysis

All data were analysed and plotted using GraphPad Prism (version 9.0.1) software. Unpaired two-tailed *t*-test or Mann-Whitney test was used to compare two datasets. Comparison of multiple datasets was carried out by ordinary two-way ANOVA with Sidak’s multiple comparisons test. Survival curves were analysed by the Log-rank (Mantel-Cox) test. TCID_50_, PFU and viral RNA copies were log-transformed before analysis. Variance between datasets was tested in all analysis. Data are presented as mean ± SEM for bar graphs and dot plots unless otherwise stated, with sample size (n) specified in figure legends. Absolute *p* values are indicated in each figure. *p* < 0.05 is considered as statistically significant.

## 3 Results

### 3.1 Three weekly doses of heat-killed *B. subtilis* spores protect mice from IAV and RSV infections

**Figure 1:**
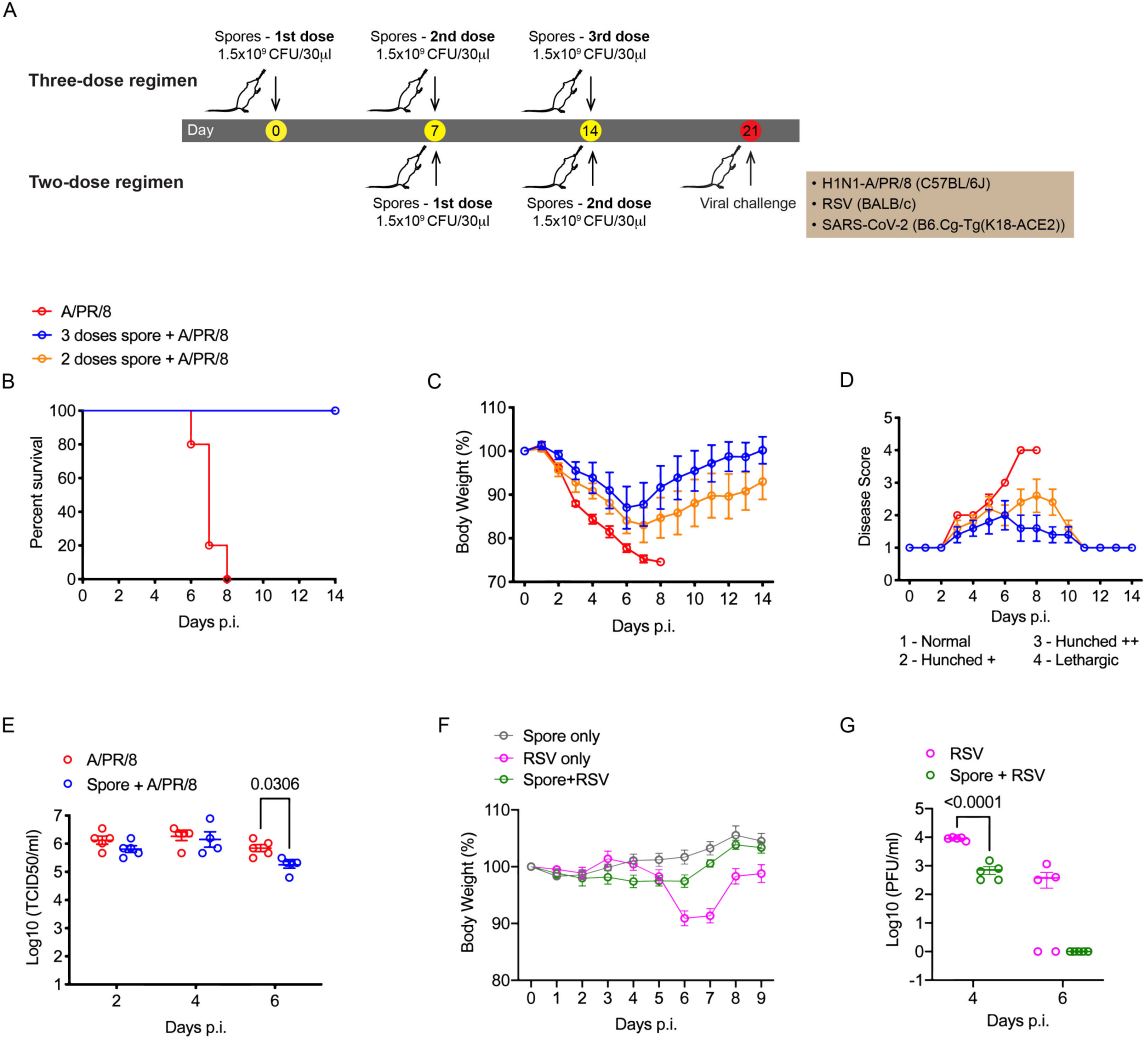
Three weekly doses of heat-killed *B*. *subtilis* protect mice from IAV and RSV infections. **(A)**, Schematic graph showing the spore dosing regimens used in this study. **(B–E)**, male C57BL/6 mice (*n* = 5 per group) were dosed intranasally with 2 or 3 doses of heat-killed spores (1 dose per week) or DPBS control, and challenged with a lethal dose of H1N1 (A/PR/8 strain, 30 PFU/50μl) influenza virus 7-days following the last nasal administration of spores. **(B)** Survival of infected mice. Mice were weighed and scored daily for 14 days following the infection. The change of body weight **(C)** was calculated as percentages to the starting weight recorded at the day of infection. **(D)** Average disease scores for each group, assessed as indicated. **(E)** Lung viral loads were determined by 50% tissue culture infectious dose (TCID_50_) at 2, 4, 6 days post the infection, *n* = 5 per group at all three timepoints. **(F, G)**, Female BALB/c mice were treated with 3 weekly doses of heat-killed spores and challenged with RSV (2.5 x 10^5^ PFU/50μl). **(F)** Change of body weight following the viral challenge (*n* = 5 per group). **(G)** Lung viral titres in infected mice measured by immunoplaque assay at indicated timepoints, n = 5 per group for each timepoint. Data shown as mean ± SEM unless otherwise stated. Ordinary two-way ANOVA with Sidak’s multiple comparisons test was used for statistical analysis of **(E, G)**. Data were from one of two independent experiments. *p* values that are lower than 0.05 are indicated.

It has been previously shown that BALB/c mice pre-treated intranasally with two doses of killed spores (on day 1 and 14 respectively) were protected from H5N1 virus infection when challenged 27-days following the last dose of spores (6). Using a different strain of *B. subtilis* (DSM 32444^K^) that had been heat-killed, we first examined whether the same two-dose regimen could protect C57BL/6 mice against lethal infection by another IAV subtype H1N1. Partial protection was observed with 60% of spore-dosed mice surviving the infection versus 0% survival for the control mice pre-treated with Dulbecco’s Phosphate Buffered Saline (DPBS) (**Supplementary Figure 1**). To optimise the efficacy of spores, we adjusted the dosing regimen by shortening the dosing interval and adding a third dose of spores to the dosing regimen. Male C57BL/6 mice were treated intranasally on day 1 & 7 (two-dose) or day 1, 7 & 14 (three-dose) with 1.5 × 10^9^ spores/dose, or the same volume of DPBS for the infection control group and challenged 7-days following the last spore dose (**Figure 1A**). Encouragingly, both dose groups exhibited 100% survival following viral challenge (**Figure 1B**). Moreover, mice receiving the three-dose regimen showed a more significant improvement in weight loss and disease symptoms (**Figures 1C, D**) together with a significant reduction of viral load in the lungs detected at day 6 post-infection (p.i.) (**Figure 1E**). These results suggest a dose-dependent protection resulting from intranasal dosing of spores against lethal H1N1 infection.

RSV is another common respiratory virus that can cause life-threatening infections in infants and elderly adults (18). It has been reported that intranasal treatment with ‘live’ *B. subtilis* spores promoted clearance of RSV in the lungs (10). We therefore thought to test the efficacy of the optimised three-dose regimen of killed spores against RSV infection. Since C57BL/6 mice are relatively resistant to RSV, BALB/c mice which are permissive for RSV replication *in vivo* were used for this pneumonia challenge model. RSV infection alone caused a significant weight loss in BALB/c mice by day 6 and 7 post-infection. In mice pre-treated with spores, no obvious weight loss was observed (**Figure 1F**) accompanied with a significant reduction of viral load in the lungs by day 4 p.i. and a complete clearance of virus by day 6 p.i. (**Figure 1G**), confirming that our spore dosing regimen was also effective against RSV.

### 3.2 Spore dosing confers protection against SARS-CoV-2 infections

Having shown that the optimised spore dosing regimen was highly effective against both H1N1 and RSV infections we next tested its efficacy against SARS-CoV-2 challenge. We used hACE2 transgenic mice expressing the humanised angiotensin-converting enzyme 2 receptor (rendering them susceptible to SARS-CoV-2 infection) (19).

**Figure 2:**
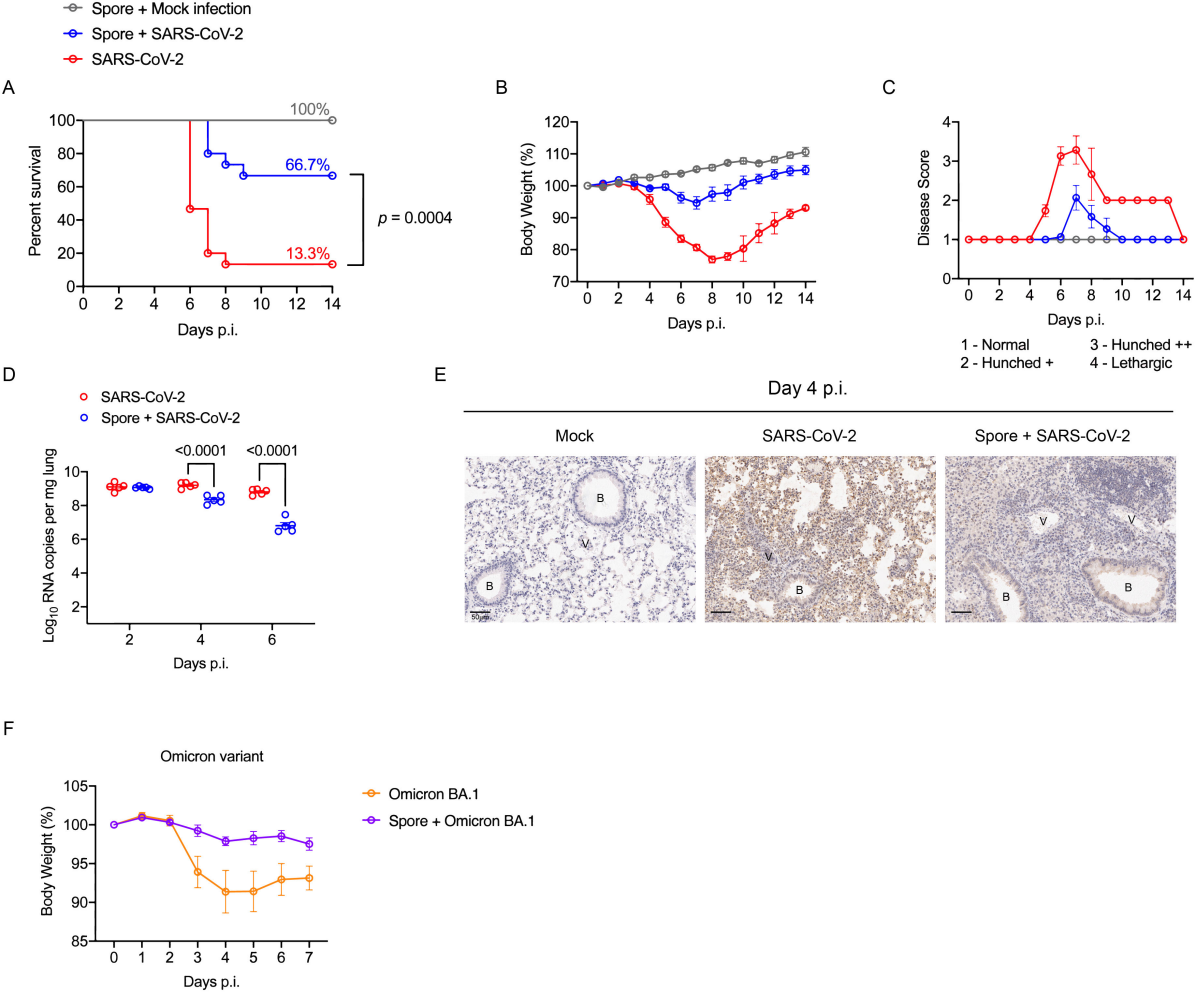
Spore dosing confers protection against SARS-CoV-2 infections. **(A–E)**, male hACE2 transgenic mice were dosed intranasally with 3 weekly doses of heat-killed spores or DPBS as controls and left for a week prior to the viral challenge with SARS-CoV-2 Beta variant. Infected mice were monitored daily for 14 days, and the disease progress was assessed by survival **(A)**, weight loss **(B)** and severity scores **(C)**. Data shown were pooled from 2 independent experiments, *n* = 5 for the mock infection group; n = 15 for SARS-CoV-2 infected groups with or without spore pre-treatment. Survival curves were compared using the Log-rank (Mantel-Cox) test. **(D)** Lung viral RNA copies in infected mice at day 2, 4, 6 post challenge, *n* = 5 per group for each timepoint. Data were analysed by ordinary two-way ANOVA with Sidak’s multiple comparisons test. **(E)** IHC staining of lung tissue sections for SARS-CoV-2 nucleocapsid, counterstained with hematoxylin (represents *n* = 3 mice per group). Scale bars, 50 μm. B, bronchiole; V, vasculature. **(F)** Male hACE2 transgenic mice were treated with 3 doses of spores and left for 27 days before infected with SARS-CoV-2 Omicron variant. The change of body weight post viral infection is presented as means ± SEM, *n* = 5 per group.

Strikingly, we observed a significant improvement in the survival of mice challenged with the SARS-CoV-2 Beta variant following our three-dose spore dosing regimen, where increased survival was accompanied by a minimum weight loss and ameliorated disease symptoms (**Figures 2A–C**). In accordance with the overall improved performance, the lung viral RNA copies in spore-dosed mice were significantly reduced by day 4 p.i. and a further reduction was detected by day 6 p.i. (**Figure 2D**). This decreased viral load was confirmed by a low viral antigen stain in the pneumocytes of spore-dosed lungs (**Figure 2E**).

To examine whether spore-mediated protection against SARS-CoV-2 could last for more than 7 days post-spore dosing, we challenged the mice 27-days after our three-dose spore dosing regimen with an Omicron variant, which at that time was the main circulating VoC (variant of concern). Mice infected with the Omicron variant did not show obvious signs of disease, such as piloerection and hunched posture, as had been observed with the Beta variant. However, a moderate weight loss was observed following the viral challenge, but not for mice that were pre-treated with spores (**Figure 2F**). In conclusion, these results suggest that spore dosing significantly protects hACE2-transgenic mice from infection caused by both Beta and Omicron variants of SARS-CoV-2. Our next challenge was to determine the immunological factors behind these protective effects.

### 3.3 Spore dosing induces immune cell recruitment and expansion of alveolar macrophages

**Figure 3:**
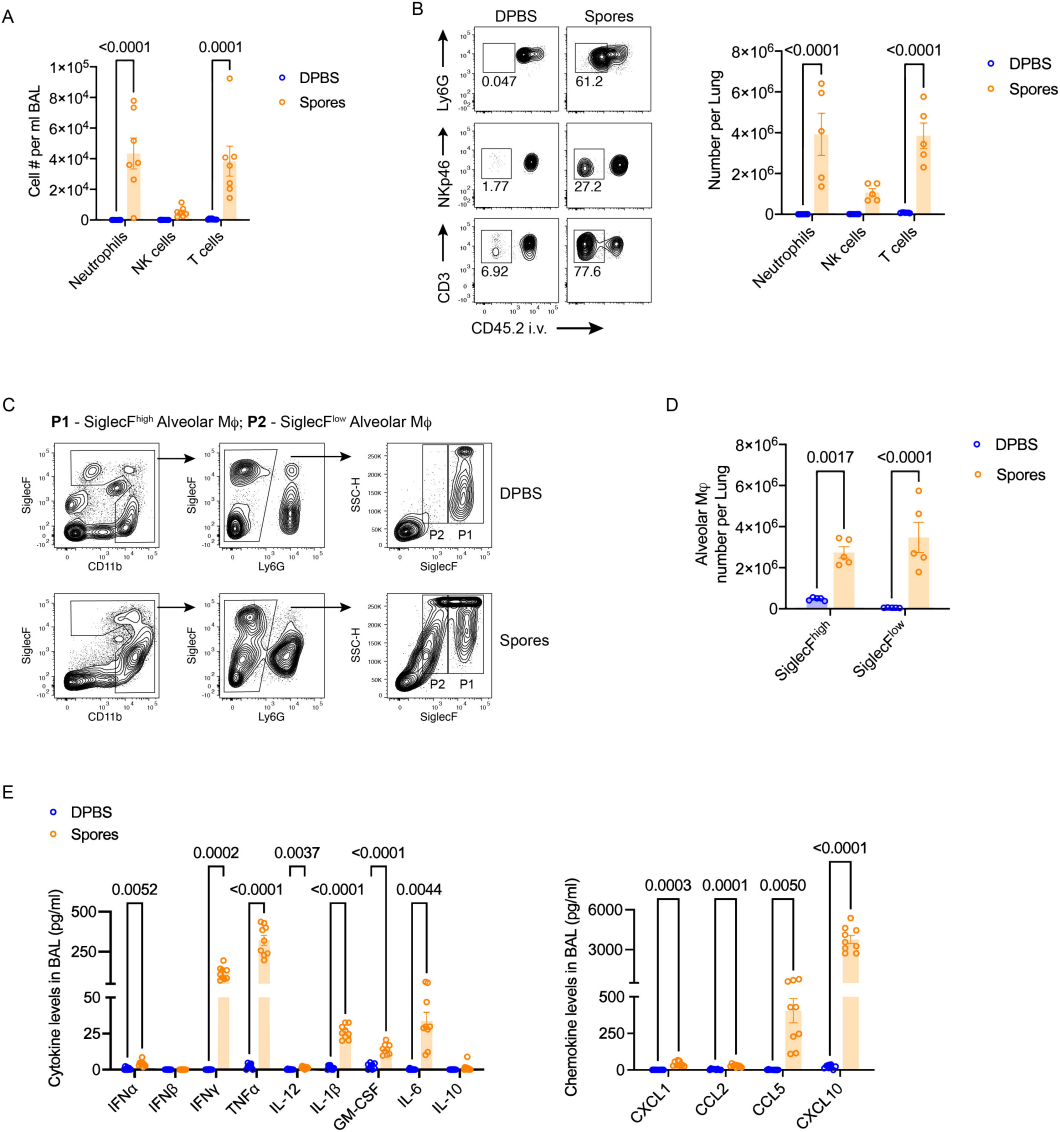
Spore dosing induces immune cell recruitment and expansion of alveolar macrophages. **(A–E)**, male C57BL/6 mice were dosed intranasally with 3 doses of heat-killed spores or control (DPBS). Immune cell recruitment in the lung was analysed 7 days after the last dose of spores. Intravascular staining with CD45.2-FITC was performed to distinguish lung parenchymal and vascular leukocytes. **(A)** Numbers of neutrophils (CD45^+^CD11b^+^Ly6G^+^), NK cells (CD45^+^CD3^-^NKp46^+^) and T cells (CD45^+^CD11b^-^CD3^+^) harvested from bronchoalveolar lavage (BAL), *n* = 7 per group pooled from 2 independent experiments. **(B)** Representative contour plots showing percentage of infiltrated neutrophils (top panel), NK cells (middle panel) and T cells (bottom panel) in the lung parenchyma (gated as CD45.2^-^ populations) following spore dosing. Numbers of these parenchymal immune cells are shown by the scattered dot plot with bars, *n* = 5 per group. **(C)** Flow cytometry gating of 2 alveolar macrophages (Mϕ) populations based on the expression levels of SiglecF in spore-treated lungs, and the numbers of SiglecF^high^ and SiglecF^low^ alveolar Mϕ are shown in **(D)**, *n* = 5 per group. Data were from one of two independent experiments. **(E)** Levels of cytokines in BAL, n = 8 for the DPBS control group and n = 9 for the spore-dosed group. Data were pooled from two independent experiment. Ordinary two-way ANOVA with Sidak’s multiple comparisons test was used for analysis.

To investigate the mechanism of spore-mediated protection, we examined leukocyte recruitment to the lungs of spore-dosed mice. Bronchioalveolar lavage (BAL) was collected to determine leukocyte populations that migrated into the airway. By intravascular staining with CD45.2 antibody, lung parenchymal (CD45.2^-^) and vasculature (CD45.2^+^) cells were distinguished to accurately define lung tissue infiltrations. Following three intranasal doses of spores, markedly higher numbers of neutrophils and T cells were recovered from the airway, and their frequency and numbers also increased significantly in the lung parenchyma (**Figures 3A, B**, **Supplementary Figure 2**). NK cell recruitment was also prominent, but the increase in number was relatively small compared to neutrophils and T cells. The results indicate that spore treatment strongly promotes leukocyte trafficking to the lungs.

Neutrophils and NK cells are important innate infiltrates that promote viral clearance at the early stages of infection and before T cell-mediated adaptive immunity has initiated (20–22). Spore recruited neutrophils and NK cells, however, this did not appear to directly contribute to viral clearance during the early stages of infection, as the lung viral loads in spore-dosed mice were not reduced till day-6 post H1N1 infection (**Figure 1D**).

Apoptosis and necrosis of alveolar epithelial cells induced directly by virus infection and dysregulated inflammation cascades are two major factors contributing to the lethality of highly virulent influenza virus and SARS-CoV-2 (23–26). Alveolar macrophages are critical immune regulators to promote viral clearance and limit tissue damage during respiratory viral infections (27–29). They can be self-renewed in physiological status or replenished by circulating monocytes when their numbers decline during infection. However, tissue-resident alveolar macrophages (TRAMs) can be distinguished from those newly recruited monocyte-derived alveolar macrophages (MoAMs) by expressing higher levels of SiglecF (30). We found that both macrophage populations, SiglecF^high^ TRAMs and SiglecF^low^ MoAMs, were significantly increased in spore-dosed lungs (**Figures 3C, D**). Hence, spores not only recruited monocytes, neutrophils, NK cells and T cells from circulation into lungs, but also promoted the expansion of lung-resident alveolar macrophages, which is key to their role in protection against respiratory viral infections. Furthermore, this enhanced activation of macrophages and other immune cells was accompanied by a significantly increased production of anti-viral cytokines, most prominently IFNγ, TNFα, CCL5 and CXCL10, in the airway of spore-treated mice (**Figure 3E**). It is worth noting that spore-treated mice also had a small increase of IFNα in the airway, but they did not produce higher levels of IFNα and IFNβ in response to the viral challenge (**Figure 3E**, **Supplementary Figure 3**).

### 3.4 Altered leukocyte trafficking and enhanced TRM induction in spore-dosed lungs following viral infection

**Figure 4:**
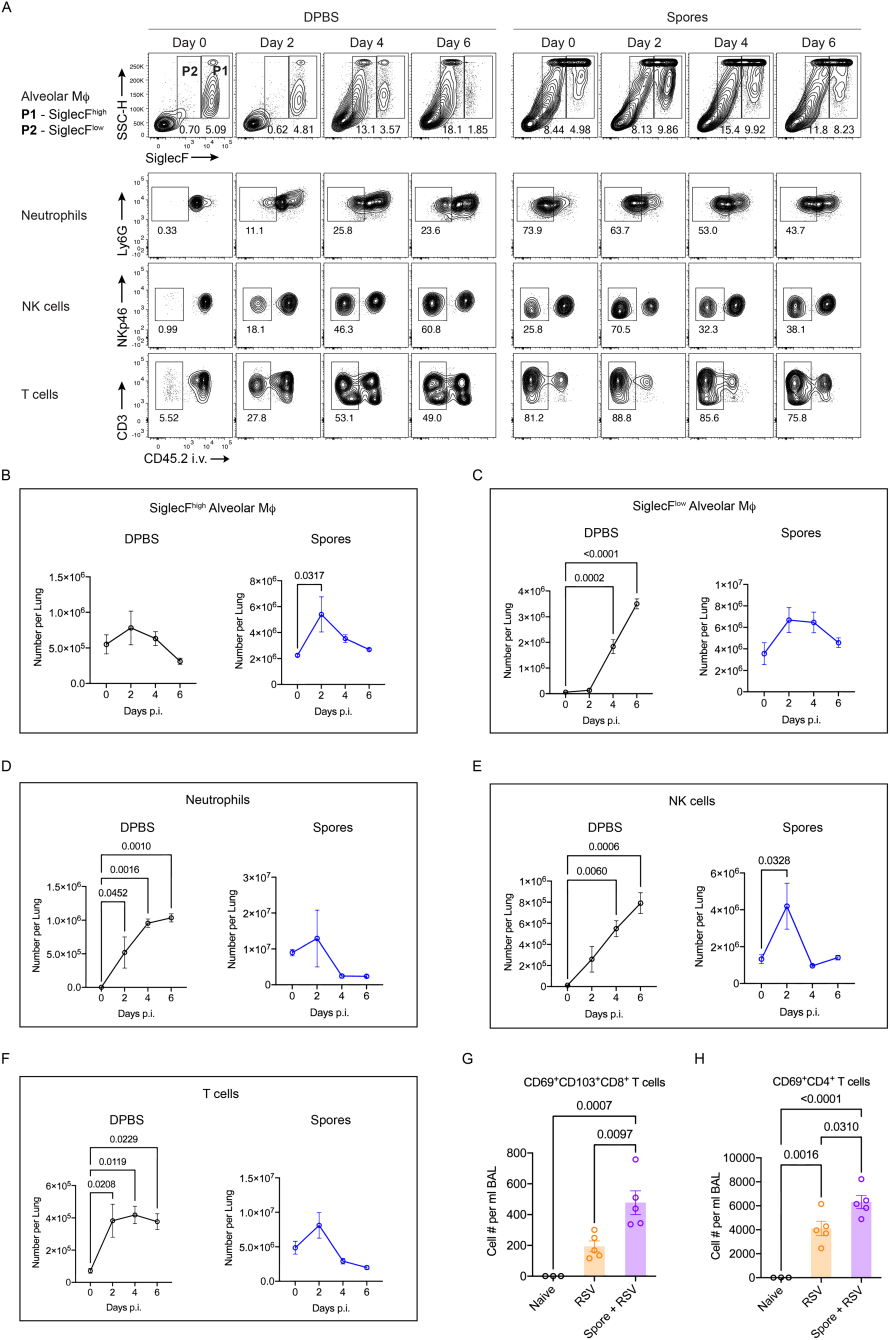
Altered leukocyte trafficking and enhanced TRM induction in spore-dosed lungs following viral infections. **(A–F)**, male C57BL/6 mice were dosed intranasally with 3 doses of heat-killed spores or controls (DPBS) and challenged by H1N1 (A/PR/8 strain) influenza virus 7 days following the last spore dose. Lung tissues were collected on day 0, 2, 4, 6 post viral challenge for analysis, *n* = 3 per group for each timepoint. Ordinary one-way ANOVA with Dunnett’s multiple comparison tests was used for analysis. Data were from one of two independent experiments. **(A)**, Representative contour plots showing the proportions of SiglecF^high^ and SiglecF^low^ alveolar Mϕ within total CD45^+^ cells, and percentages of infiltrated neutrophils, NK cells and T cells in the lung parenchyma (gated as CD45.2^-^ populations) following viral challenge. **(B, C)** Numbers of SiglecF^high^ **(B)** and SiglecF^low^ **(C)** alveolar Mϕ over the infection course in spore pre-treated and DPBS pre-treated control mice. **(D–F)** Numbers of infiltrated (CD45.2^-^ parenchymal cells) neutrophils **(D)**, NK cells **(E)** and T cells **(F)** over the infection course. **(G, H)** Female BALB/c mice were treated with 3 weekly doses of heat-killed spores or control treated with DPBS and challenged by RSV. Numbers of tissue resident memory CD8^+^ T cells (CD69^+^CD103^+^, **G**) and CD4^+^ T cells (CD69^+^, **H**) in Bronchoalveolar lavage (BAL) harvested at day 4 post viral challenge. *n* = 3 for the Naïve and *n* = 5 per group for the RSV infected. Data were from one of two independent experiments and analysed using ordinary two-way ANOVA with Sidak’s multiple comparisons test.

We then examined the effect of spore dosing on leukocyte trafficking in the context of lethal H1N1 infection. Prior to viral challenge and at day 2, 4 & 6 p.i., mouse lungs were harvested to analyse parenchymal leukocyte infiltration. Spore treatment led to a further increase in TRAMs at day 2 p.i. (**Figures 4A, B**), suggesting enhanced self-renewal of spore-activated TRAMs during early exposure to highly pathogenic virus. Whilst the viral infection induced a marked recruitment of MoAMs, neutrophils and NK cells to the lungs of DPBS-treated control mice, mice pre-treated with spores had higher basal levels of these innate immune cells in their lungs but a further recruitment in response to the infection was only seen for NK cells at day 2 p.i. (**Figures 4A–E**). The significant increase in total T cells by day 2 p.i. was also not seen in spore-dosed lungs (**Figure 4F**). In general, pre-treating with spores appeared to dampen the inflammatory responses elicited by highly virulent H1N1 virus, suggesting that spores may prevent the development of severe viral pneumonia by inducing immune tolerance and thereby limiting inflammation associated tissue damage. Furthermore, intranasal boosting with spores significantly increased the numbers of both CD8^+^ and CD4^+^ tissue resident memory T cells (TRMs) following RSV challenge (**Figures 4G–H**). These results show that spores can regulate both innate and adaptive immunity for protection against viral infections.

### 3.5 Intranasal spore dosing boosts antibody production against SARS-CoV-2 Spike protein

**Figure 5:**
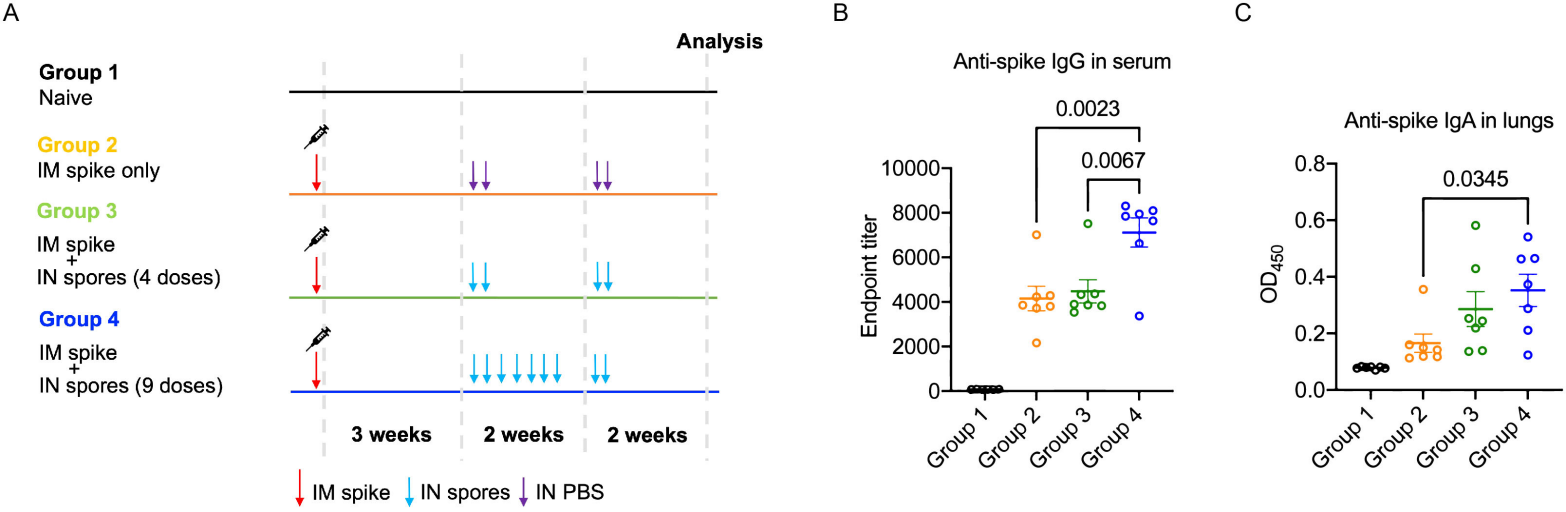
Intranasal spore dosing boosts antibody production against SARS-CoV-2 spike protein. Mice were intranasally administrated with killed spores following a parenteral prime. The dosing regimens for the four study groups (*n* = 7 per group, pooled from two independent experiments) are shown in **(A)**. Group 1 was a naïve control group with animals receiving no treatment. Groups 2-4 all received an intra-muscular prime of recombinant Spike protein (5 μg). Three weeks later Groups 3 and 4 were dosed intranasally with spores using either 4 doses spread over 2-weeks (Group 3; days 21, 23, 35 & 37) or 9 consecutive doses (Group 4; days 21, 23, 25, 27, 29, 31, 33, 35 & 37). Group 2 received PBS as controls (days 21, 23, 25 & 37). Anti-Spike IgG in serum **(B)** and anti-Spike SIgA in lungs **(C)** was measured at day 49 and day 50 respectively. Data were analysed using ordinary two-way ANOVA with Tukey’s multiple comparisons test.

We demonstrated that intranasal administration of spores protected against multiple viral infections, and this was associated with regulated inflammatory leukocyte trafficking and potentially might also involve enhanced TRM activation. We then further assessed their potential as a nasal booster following parenteral vaccination by first parenterally immunising mice with SARS-CoV-2 recombinant Spike protein, and then boosting with 4 or 9 i.n. doses of spores three weeks after (**Figure 5A**). We detected significantly higher levels of serum IgG and pulmonary IgA against the Spike protein following 9 doses of spores (**Figures 5B, C**), suggesting spores have the potential to ‘pull’ pre-existing immune memory to mucosal sites if used in conjunction with current COVID-19 vaccines.

## 4 Discussion

We show in this study that intranasal dosing with *B. subtilis* spores confers protection against a number of common respiratory viral infections, including the highly virulent H1N1 influenza virus, Beta and Omicron variants of SARS-CoV-2 and finally RSV. This spore-induced heterologous protection is associated with expanded alveolar macrophage populations, controlled leukocyte trafficking and enhanced TRM induction when challenged with virus. The ability of spores to enhance antibody production when administrated as a nasal booster following parenteral immunisation further demonstrates their significant potential as a mucosal vaccine.

Both TRAMs and MoAMs were markedly increased in spore treated lungs (**Figures 3C, D**), suggesting that spores not only activate the recruitment of monocytes from the periphery but also promote the proliferation of TRAMs, which may be mediated by enhanced GM-CSF (granulocyte-macrophage colony-stimulating factor) production by lung epithelial cells (31). The protective role of TRAMs in IAV infection has been well established in previous studies (20, 27, 29). Transgenic mice that over-express GM-CSF have increased numbers of TRAMs in the lung, and hence are resistant to IAV infections. However, depleting TRAMs in these mice in the first three days following H1N1 infection lead to lethal outcomes (32), suggesting that an enhanced TRAM response in the early stages of infection is a key factor in prevention of poor prognosis. Our results show that TRAMs were further increased by day-2 post H1N1 infection in spore-treated lungs, which may be critical for protection against both IAV and SARS-CoV-2. In addition, spore-activated alveolar macrophages have been shown to enhance anti-viral function against RSV (10). Clearly, the duration of induced TRAM levels resulting from spore dosing is key to the efficacy of protection. We show here that spore-mediated protection against SARS-CoV-2 persisted for at least 27 days following the last dose of spores. Further studies will be required to determine the maximum protective duration following spore dosing and its association with the effect on TRAM renewal and function. Enhanced macrophage activation against pathogens is also a sign of trained immunity by previous exposure to unrelated stimulations. It would be interesting to explore whether spore treatment is able to reprogramme TRAM, making them more efficient at clearing invading pathogens.

Uncontrolled leukocyte trafficking elicited by viral infection is a major cause of tissue pathology and can lead to systemic dissemination of pathogens (33). Excessive neutrophil infiltration has frequently been observed in severe cases of both COVID-19 and influenza pneumonia (33–35). Enhanced recruitment of highly inflammatory monocytes also appears to directly correlate with COVID-19 severity (36). Although spores by themselves can activate the trafficking of these innate immune cells directly into lungs, recruitment in response to viral infection in spore-dosed lungs is attenuated (**Figure 4**). This may correlate with the highly expanded population of TRAMs in spore-dosed lungs, that are able to modulate inflammatory immune responses and limit tissue damage, in addition to their direct anti-viral function (28, 37). It has been suggested that *Lactobacillus* species increase resistance to severe viral infections (pneumonia virus of mice (PVM) and H1N1 influenza) through attenuating anti-viral inflammatory responses (38–40). This protection mechanism may also apply to *B. subtilis* spores. The ability to prevent excessive anti-viral inflammatory responses may be a common trait for the heterologous immunity induced by probiotic and commensal microorganisms.

Apart from modulating innate immunity, nasal dosing with spores was also able to promote tissue-resident memory T cell responses, as well as enhancing anti-spike antibody production following intramuscular immunisation with SARS-CoV-2 Spike protein (**Figures 4**, **5**). Whether the enhancement of adaptive immunity correlates with the activation of alveolar macrophages and other innate immune cells will require further investigation. However, we cannot exclude potential cross-reactivity between the *bacillus* spores and viruses. A human study found SARS-CoV-2-specific T cells in pre-pandemic adult blood which can cross-react to commensal bacterial antigens (41). It is possible that *bacillus* spores can induce antibodies and/or T cells that are able to cross-react to influenza virus/SARS-CoV-2/RSV. It would be interesting to explore whether viral antigen-specific T and B cells are induced by intranasal dosing of spores in future studies. What is clear though is that by simultaneously boosting anti-viral antibody responses and expanding protective alveolar macrophage populations, spores used in conjunction (as adjuncts) with vaccines could confer stronger resistance to highly virulent pneumotropic viruses by accelerating viral clearance and ameliorating collateral lung injury. This further highlights the potential of spores for development as a universal anti-viral nasal boost.

There are a multitude of advantages for utilising spores as part of a Pan-Pneumovirus vaccine. First, spores are intrinsically robust and able to withstand desiccation and exposure to temperature extremes (-20°C - 120°C) (42). As such, they can be stored and transported in liquid or solid form without the need of a cold chain. Second, as shown here, the spore exerts its immunogenic effect in an inactive form, that is, there is no requirement for a live spore. *B. subtilis* is of course recognised as non-pathogenic, and it has been reported that nasal spray of probiotic live bacillus spores (mixed *B. subtilis* and *B. clausii*) was safe in humans (11). However, it is possible that live spores could germinate and proliferate within the airways or lungs which can be a potential issue. The use of killed spores has no such concerns, and they can be rapidly cleared from the lungs and airways when assessed in mice (43). More importantly, intranasal administration of the inactivated *B. subtilis* DSM 32444 spores used in this study has been tested in human volunteers, with no adverse events observed (44). Taken together, inactivated *B. subtilis* spores are well tolerated by both animals and humans which makes them an attractive mucosal vaccine candidate in humans.

Further studies will be necessary to fully understand the exact mechanisms underlying the protective effects of spores, including the role of specific immune cell populations and the impact of spore dosing on immune memory. Additionally, the potential of this treatment to be used in combination with other antiviral therapies should be explored. Overall, the results of this study provide a promising avenue for the development of novel antiviral vaccines and therapies and represent an important step towards the prevention and treatment of respiratory viral infections.

## Data Availability

The original contributions presented in the study are included in the article/**Supplementary Material**. Further inquiries can be directed to the corresponding authors.

## References

[1] Troeger C Blacker B Khalil IA Rao PC Cao J Zimsen SRM. Estimates of the global, regional, and national morbidity, mortality, and aetiologies of lower respiratory infections in 195 countries, 1990–2016: a systematic analysis for the Global Burden of Disease Study 2016. Lancet Infect Diseases. (2018) 18:1191–210. doi: 10.1016/S1473-3099(18)30310-4 PMC6202443 30243584

[2] Nazareth J Pan D Martin CA Barr I Sullivan SG Stephenson I. Is the UK prepared for seasonal influenza in 2022-23 and beyond? Lancet Infect Dis. (2022) 22:1280–1. doi: 10.1016/S1473-3099(22)00503-5 PMC9348822 35932777

[3] Nicholson WL. Roles of Bacillus endospores in the environment. Cell Mol Life Sci. (2002) 59:410–6. doi: 10.1007/s00018-002-8433-7 PMC11337551 11964119

[4] Hong HA Hitri K Hosseini S Kotowicz N Bryan D Mawas F. Mucosal antibodies to the C terminus of toxin A prevent colonization of *clostridium difficile* . Infect Immun. (2017) 85(4):e01060-16. doi: 10.1128/IAI.01060-16 28167669 PMC5364299

[5] Duc le H Hong HA Fairweather N Ricca E Cutting SM. Bacterial spores as vaccine vehicles. Infect Immun. (2003) 71:2810–8. doi: 10.1128/IAI.71.5.2810-2818.2003 PMC153275 12704155

[6] Song M Hong HA Huang J-M Colenutt C Khang DD Nguyen TVA. Killed Bacillus subtilis spores as a mucosal adjuvant for an H5N1 vaccine. Vaccine. (2012) 30:3266–77. doi: 10.1016/j.vaccine.2012.03.016 22446640

[7] Welsh RM Che JW Brehm MA Selin LK. Heterologous immunity between viruses. Immunol Rev. (2010) 235:244–66. doi: 10.1111/j.0105-2896.2010.00897.x PMC2917921 20536568

[8] Walk J de Bree LCJ Graumans W Stoter R van Gemert GJ van-de-Vegte-Bolmer M. Outcomes of controlled human malaria infection after BCG vaccination. Nat Commun. (2019) 10:874. doi: 10.1038/s41467-019-08659-3 30787276 PMC6382772

[9] Kleinnijenhuis J Quintin J Preijers F Joosten LA Ifrim DC Saeed S. Bacille Calmette-Guerin induces NOD2-dependent nonspecific protection from reinfection via epigenetic reprogramming of monocytes. Proc Natl Acad Sci U S A. (2012) 109:17537–42. doi: 10.1073/pnas.1202870109 PMC3491454 22988082

[10] Hong JE Kye YC Park SM Cheon IS Chu H Park BC. Alveolar macrophages treated with bacillus subtilis spore protect mice infected with respiratory syncytial virus A2. Front Microbiol. (2019) 10:447. doi: 10.3389/fmicb.2019.00447 30930867 PMC6423497

[11] Tran TT Phung TTB Tran DM Bui HT Nguyen PTT Vu TT. Efficient symptomatic treatment and viral load reduction for children with influenza virus infection by nasal-spraying Bacillus spore probiotics. Sci Rep. (2023) 13:14789. doi: 10.1038/s41598-023-41763-5 37684332 PMC10491672

[12] Morton DB Griffiths PH. Guidelines on the recognition of pain, distress and discomfort in experimental animals and an hypothesis for assessment. Vet Rec. (1985) 116:431–6. doi: 10.1136/vr.116.16.431 3923690

[13] Van Hoecke L Job ER Saelens X Roose K. Bronchoalveolar lavage of murine lungs to analyze inflammatory cell infiltration. J Visualized Experiments. (2017), (123):55398. doi: 10.3791/55398 PMC5607888 28518083

[14] Xu R Jacques LC Khandaker S Beentjes D Leon-Rios M Wei X. TNFR2(+) regulatory T cells protect against bacteremic pneumococcal pneumonia by suppressing IL-17A-producing gammadelta T cells in the lung. Cell Rep. (2023) 42:112054. doi: 10.1016/j.celrep.2023.112054 36724074

[15] Karakus U Crameri M Lanz C Yángüez E. Propagation and titration of influenza viruses. Methods Mol Biol. (2018) 1836:59–88. doi: 10.1007/978-1-4939-8678-1_4 30151569

[16] Currie SM Findlay EG McHugh BJ Mackellar A Man T Macmillan D. The human cathelicidin LL-37 has antiviral activity against respiratory syncytial virus. PloS One. (2013) 8:e73659. doi: 10.1371/journal.pone.0073659 24023689 PMC3758310

[17] Katsande PM Fernandez-Bastit L Ferreira WT Vergara-Alert J Hess M Lloyd-Jones K. Heterologous systemic prime-intranasal boosting using a spore SARS-coV-2 vaccine confers mucosal immunity and cross-reactive antibodies in mice as well as protection in hamsters. Vaccines (Basel). (2022) 10:1900. doi: 10.3390/vaccines10111900 36366408 PMC9692796

[18] Meng J Stobart CC Hotard AL Moore ML. An overview of respiratory syncytial virus. PloS pathogens. (2014) 10:e1004016. doi: 10.1371/journal.ppat.1004016 24763387 PMC3999198

[19] Winkler ES Bailey AL Kafai NM Nair S McCune BT Yu J. SARS-CoV-2 infection of human ACE2-transgenic mice causes severe lung inflammation and impaired function. Nat Immunol. (2020) 21:1327–35. doi: 10.1038/s41590-020-0778-2 PMC7578095 32839612

[20] Tumpey TM Garcia-Sastre A Taubenberger JK Palese P Swayne DE Pantin-Jackwood MJ. Pathogenicity of influenza viruses with genes from the 1918 pandemic virus: functional roles of alveolar macrophages and neutrophils in limiting virus replication and mortality in mice. J Virol. (2005) 79:14933–44. doi: 10.1128/JVI.79.23.14933-14944.2005 PMC1287592 16282492

[21] Stein-Streilein J Guffee J. *In vivo* treatment of mice and hamsters with antibodies to asialo GM1 increases morbidity and mortality to pulmonary influenza infection. J Immunol. (1986) 136:1435–41. doi: 10.4049/jimmunol.136.4.1435 3944461

[22] Dunning J Thwaites RS Openshaw PJM. Seasonal and pandemic influenza: 100 years of progress, still much to learn. Mucosal Immunol. (2020) 13:566–73. doi: 10.1038/s41385-020-0287-5 PMC7223327 32317736

[23] Brandes M Klauschen F Kuchen S Germain RN. A systems analysis identifies a feedforward inflammatory circuit leading to lethal influenza infection. Cell. (2013) 154:197–212. doi: 10.1016/j.cell.2013.06.013 23827683 PMC3763506

[24] Atkin-Smith GK Duan M Chen W Poon IKH. The induction and consequences of Influenza A virus-induced cell death. Cell Death Dis. (2018) 9:1002. doi: 10.1038/s41419-018-1035-6 30254192 PMC6156503

[25] D’Agnillo F Walters KA Xiao Y Sheng ZM Scherler K Park J. Lung epithelial and endothelial damage, loss of tissue repair, inhibition of fibrinolysis, and cellular senescence in fatal COVID-19. Sci Transl Med. (2021) 13:eabj7790. doi: 10.1126/scitranslmed.abj7790 34648357 PMC11000440

[26] Montazersaheb S Hosseiniyan Khatibi SM Hejazi MS Tarhriz V Farjami A Ghasemian Sorbeni F. COVID-19 infection: an overview on cytokine storm and related interventions. Virol J. (2022) 19:92. doi: 10.1186/s12985-022-01814-1 35619180 PMC9134144

[27] Wong CK Smith CA Sakamoto K Kaminski N Koff JL Goldstein DR. Aging impairs alveolar macrophage phagocytosis and increases influenza-induced mortality in mice. J Immunol. (2017) 199:1060–8. doi: 10.4049/jimmunol.1700397 PMC5557035 28646038

[28] Duan M Hibbs ML Chen W. The contributions of lung macrophage and monocyte heterogeneity to influenza pathogenesis. Immunol Cell Biol. (2017) 95:225–35. doi: 10.1038/icb.2016.97 27670791

[29] Schneider C Nobs SP Heer AK Kurrer M Klinke G van Rooijen N. Alveolar macrophages are essential for protection from respiratory failure and associated morbidity following influenza virus infection. PloS pathogens. (2014) 10:e1004053. doi: 10.1371/journal.ppat.1004053 24699679 PMC3974877

[30] Misharin AV Morales-Nebreda L Mutlu GM Budinger GR Perlman H. Flow cytometric analysis of macrophages and dendritic cell subsets in the mouse lung. Am J Respir Cell Mol Biol. (2013) 49:503–10. doi: 10.1165/rcmb.2013-0086MA PMC3824047 23672262

[31] Suzuki T McCarthy C Carey BC Borchers M Beck D Wikenheiser-Brokamp KA. Increased pulmonary GM-CSF causes alveolar macrophage accumulation. Mechanistic implications for desquamative interstitial pneumonitis. Am J Respir Cell Mol Biol. (2020) 62:87–94. doi: 10.1165/rcmb.2018-0294OC 31310562 PMC6938130

[32] Huang F-F Barnes PF Feng Y Donis R Chroneos ZC Idell S. GM-CSF in the lung protects against lethal influenza infection. Am J Respir Crit Care Med. (2011) 184:259–68. doi: 10.1164/rccm.201012-2036OC PMC6938174 21474645

[33] Alon R Sportiello M Kozlovski S Kumar A Reilly EC Zarbock A. Leukocyte trafficking to the lungs and beyond: lessons from influenza for COVID-19. Nat Rev Immunol. (2021) 21:49–64. doi: 10.1038/s41577-020-00470-2 33214719 PMC7675406

[34] Xu T Qiao J Zhao L Wang G He G Li K. Acute respiratory distress syndrome induced by avian influenza A (H5N1) virus in mice. Am J Respir Crit Care Med. (2006) 174:1011–7. doi: 10.1164/rccm.200511-1751OC 16917113

[35] McKenna E Wubben R Isaza-Correa JM Melo AM Mhaonaigh AU Conlon N. Neutrophils in COVID-19: not innocent bystanders. Front Immunol. (2022) 13:864387. doi: 10.3389/fimmu.2022.864387 35720378 PMC9199383

[36] Liao M Liu Y Yuan J Wen Y Xu G Zhao J. Single-cell landscape of bronchoalveolar immune cells in patients with COVID-19. Nat Med. (2020) 26:842–4. doi: 10.1038/s41591-020-0901-9 32398875

[37] Uderhardt S Martins AJ Tsang JS Lammermann T Germain RN. Resident macrophages cloak tissue microlesions to prevent neutrophil-driven inflammatory damage. Cell. (2019) 177:541–55.e17. doi: 10.1016/j.cell.2019.02.028 30955887 PMC6474841

[38] Park MK Ngo V Kwon YM Lee YT Yoo S Cho YH. Lactobacillus plantarum DK119 as a probiotic confers protection against influenza virus by modulating innate immunity. PloS One. (2013) 8:e75368. doi: 10.1371/journal.pone.0075368 24124485 PMC3790790

[39] Garcia-Crespo KE Chan CC Gabryszewski SJ Percopo CM Rigaux P Dyer KD. Lactobacillus priming of the respiratory tract: Heterologous immunity and protection against lethal pneumovirus infection. Antiviral Res. (2013) 97:270–9. doi: 10.1016/j.antiviral.2012.12.022 PMC3608699 23274789

[40] Gabryszewski SJ Bachar O Dyer KD Percopo CM Killoran KE Domachowske JB. Lactobacillus-mediated priming of the respiratory mucosa protects against lethal pneumovirus infection. J Immunol. (2011) 186:1151–61. doi: 10.4049/jimmunol.1001751 PMC3404433 21169550

[41] Bartolo L Afroz S Pan YG Xu R Williams L Lin CF. SARS-CoV-2-specific T cells in unexposed adults display broad trafficking potential and cross-react with commensal antigens. Sci Immunol. (2022) 7:eabn3127. doi: 10.1126/sciimmunol.abn3127 35857619 PMC9348748

[42] Nicholson WL Munakata N Horneck G Melosh HJ Setlow P. Resistance of Bacillus endospores to extreme terrestrial and extraterrestrial environments. Microbiol Mol Biol Rev. (2000) 64:548–72. doi: 10.1128/MMBR.64.3.548-572.2000 PMC99004 10974126

[43] Reljic R Sibley L Huang JM Pepponi I Hoppe A Hong HA. Mucosal vaccination against tuberculosis using inert bioparticles. Infection immunity. (2013) 81:4071–80. doi: 10.1128/IAI.00786-13 PMC3811844 23959722

[44] Ho YT Hynes D Martina Y Love B Horwell E Xu R. Intranasal administration of DSM 32444 Bacillus subtilis spores: safety and tolerability. J Med Microbiol. (2024) 73(7). doi: 10.1099/jmm.0.001845 38963177

