# Supplementary material for: Nasal delivery of killed Bacillus subtilis spores protects against influenza, RSV and SARS-CoV-2
Source: Front Immunol. 2025 Apr 2;16:1501907. doi: 10.3389/fimmu.2025.1501907 (PMC12000887; doi:10.3389/fimmu.2025.1501907)
Supplement: Supplementary file 1 [file DataSheet1.pdf]

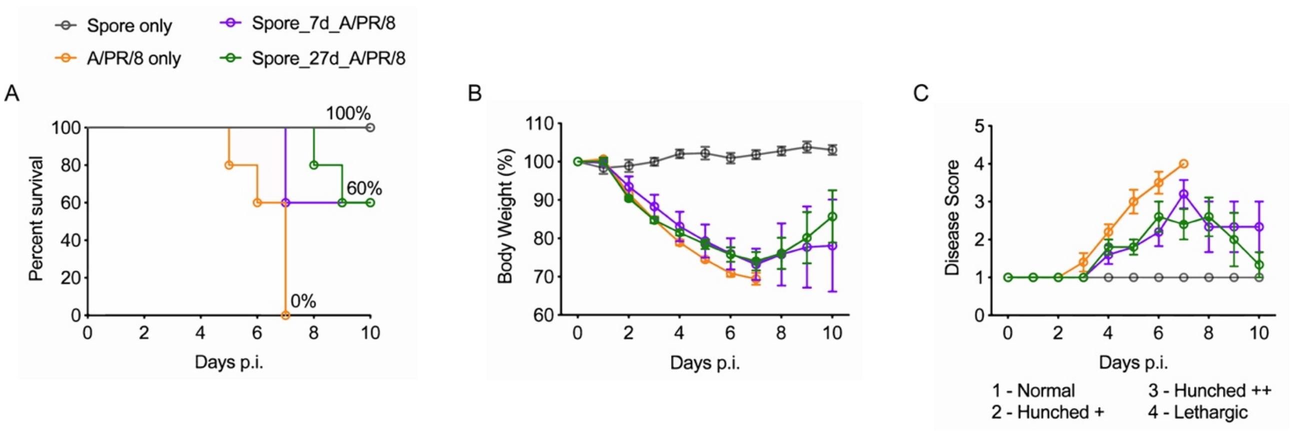

Supplementary Fig. 1

Female C57BL/6 mice (*n* = 5 per group) were dosed intranasally with heat-killed spores (1.5×10^9 CFU/30μl) or DPBS control on day 1 and 14 respectively. 7 or 27 days post the 2nd dose of spores, mice were challenged with H1N1 (A/PR/8 strain) influenza virus. (**A**) Survival of infected mice. The change of body weight (**B**) was calculated as percentages to the starting weight recorded at the day of infection. (**C**) Average disease scores for each group, assessed as indicated.

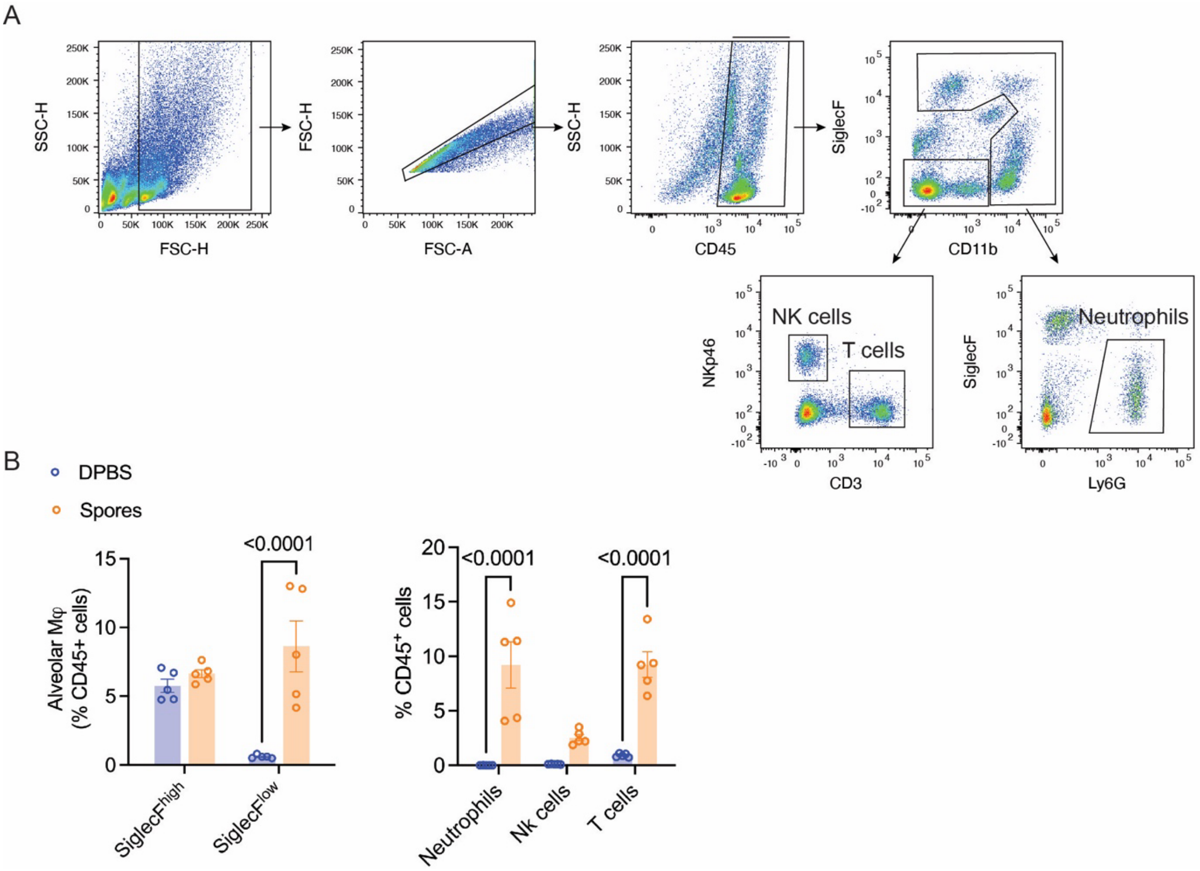

Supplementary Fig. 2

Male C57BL/6 mice were dosed intranasally with 3 doses of heat-killed spores or control (DPBS). Immune cell recruitment in the lung was analysed 7 days after the last dose of spores. (**A**) Flow cytometry gating of neutrophils ($CD45^+CD11b^+Ly6G^+$), NK cells ($CD45^+CD3^-NKp46^+$) and T cells ($CD45^+CD11b^-CD3^+$) in isolated lung cells. (**B**) The percentages of alveolar macrophages (Mϕ) populations and infiltrated neutrophils, NK cells and T cells in the lung parenchyma (gated as $CD45.2^-$) of DPBS and spore dosed mice. Data were analysed using ordinary two-way ANOVA with Sidak's multiple comparisons test, *n* =5 per group, from one of two independent experiments.

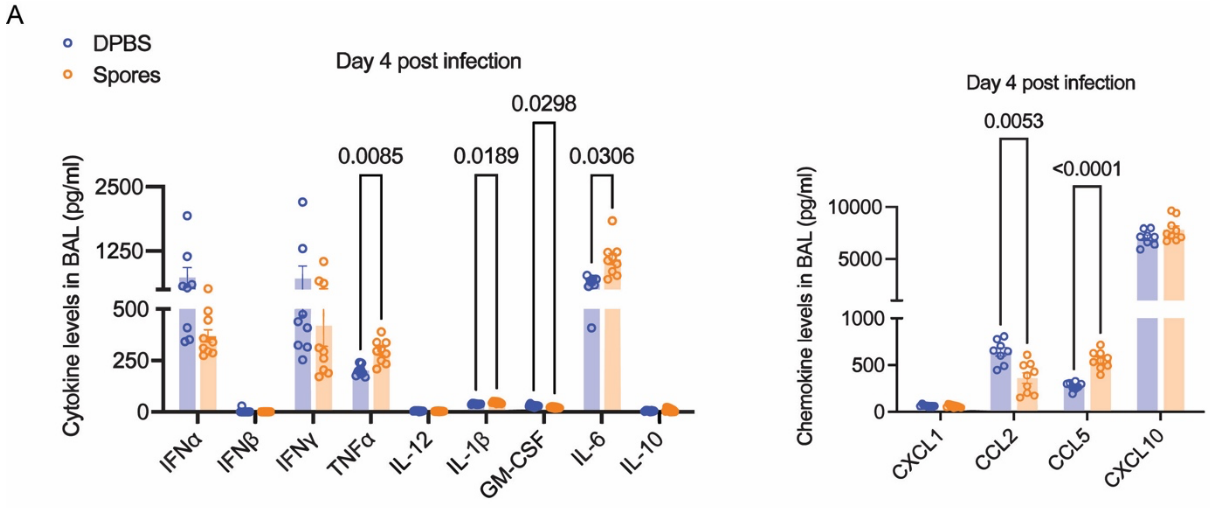

Supplementary Fig. 3

Male C57BL/6 mice were dosed intranasally with 3 doses of heat-killed spores or controls (DPBS) and challenged by H1N1 (A/PR/8 strain) influenza virus 7 days following the last spore dose. Bronchoalveolar lavage (BAL) was collected on day 4 post viral challenge to determine cytokine levels by cytometric bead array. n = 8 for the DPBS control group and n = 9 for the spore-dosed group. Data were pooled from two independent experiment. Ordinary two-way ANOVA with Sidak's multiple comparisons test was used for analysis.
